# Bipolar lophotrichous *Helicobacter suis* combine extended and wrapped flagella bundles to exhibit multiple modes of motility

**DOI:** 10.1038/s41598-018-32686-7

**Published:** 2018-09-26

**Authors:** Maira A. Constantino, Mehdi Jabbarzadeh, Henry C. Fu, Zeli Shen, James G. Fox, Freddy Haesebrouck, Sara K. Linden, Rama Bansil

**Affiliations:** 1 Boston University, Boston, MA 02215 USA; 2 University of Utah, Salt Lake City, Utah USA; 3 Massachusetts Institute of Technology, Cambridge, MA 02138 USA; 4 Ghent University, Ghent, Belgium; 5 University of Gothenburg, Gothenburg, Sweden

## Abstract

The swimming strategies of unipolar flagellated bacteria are well known but little is known about how bipolar bacteria swim. Here we examine the motility of *Helicobacter suis*, a bipolar gastric-ulcer-causing bacterium that infects pigs and humans. Phase-contrast microscopy of unlabeled bacteria reveals flagella bundles in two conformations, extended away from the body (E) or flipped backwards and wrapped (W) around the body. We captured videos of the transition between these two states and observed three different swimming modes in broth: with one bundle rotating wrapped around the body and the other extended (EW), both extended (EE), and both wrapped (WW). Only EW and WW modes were seen in porcine gastric mucin. The EW mode displayed ballistic trajectories while the other two displayed superdiffusive random walk trajectories with slower swimming speeds. Separation into these two categories was also observed by tracking the mean square displacement of thousands of trajectories at lower magnification. Using the Method of Regularized Stokeslets we numerically calculate the swimming dynamics of these three different swimming modes and obtain good qualitative agreement with the measurements, including the decreased speed of the less frequent modes. Our results suggest that the extended bundle dominates the swimming dynamics.

## Introduction

The ability of bacteria to explore their surroundings is very important for survival^1^. In order to reach nutritious regions or to escape harsh environments and toxins, different chemotactic bacteria species adopt different swimming strategies to swim towards an attractant or away from a repellent. The strategies of unipolar flagellated bacteria (one flagellum or multiple flagella situated at one end of the body) or peritrichous bacteria (multiple flagella distributed uniformly around the body) are very well known. The most commonly known swimming strategy is run-and–tumble^2,3^, as shown in *E*. *coli*, a peritrichous bacterium with a ~2 μm rod-shaped body. When it swims forward, the flagella rotate counter-clockwise, forming a coherent bundle at one pole of the body. If one or more flagellum rotates clockwise, the tuft unbundles, the bacterium stops translating temporarily, and tumbles^4^. When the flagella resume counter-clockwise rotation, they reform a bundle and the bacterium swims in a new random direction with an average turning angle of ~70°. Unipolar monotrichous bacteria (single flagellum at one end of the body), like *Pseudoalteromonas haloplanktis*^5,6^ as well as unipolar lophotrichous bacteria (multiple flagella arranged at one end of the body), like the human stomach pathogen *Helicobacter pylori*^7,8^ or the soil bacteria *Pseudomonas putida*^9–11^ adopt a different swimming strategy, namely run-and-reverse swimming. They can either swim as a pusher, with the bundle extended lagging behind the body, or as a puller, with the motor rotating the opposite way, and with the bundle located in front of the body. Modifications of this basic run-reverse strategy such as run-reverse-flick strategy are seen in *Vibrio alginolyticus*^12,13^. In run-reverse-flick, the bacterium performs a forward run as a pusher, reverses direction swimming as a puller and when it switches back to swim forward, the flagellum flicks to select a new direction. In our recent work^14^ we imaged the rotation of *H*. *pylori* while swimming forward, reorienting, as well as reversing, and we were able to observe the counter rotation of the cell body reversing on a reversal, consistent with a scenario in which during both pushing (forward motion) and pulling (backwards motion) the propulsive flagella have the same left-handedness.

Many flagellated bacteria are bipolar: amphitrichous bacteria such as the dolphin stomach pathogen *Helicobacter cetorum*, the human pathogen *Campylobacter jejuni*, and the freshwater magnetotactic bacteria *Magnetospirillum magneticum* all have a single flagella at each end of the body, while *Helicobacter suis* has multiple flagella at each pole. However much less is known about the swimming strategies of bipolar bacteria as opposed to unipolar bacteria. The first published study is from Murat *et al*.^15^ in 2015 who examined how bipolar flagellated bacteria swim by labeling the flagella of the amphitrichous bacteria *M*. *magneticum* and imaging it under an externally applied magnetic field. This bacterium contains a chain of magnetic particles on the surface of its helical body, which confers a magnetic moment to the cell, hence it orients itself in the presence of an external magnetic field. Due to the fast rotating flagella compared to the exposure time of the camera (80 to 100 ms), they were not able to capture the flagella while in motion, however they observed two different patterns of fluorescence likely corresponding to flagella rotating in opposite directions (Figs 4 and 5 from Murat *et al*.^15^). One pattern was named tuft and corresponds to a flagellum extended away from the body, as commonly seen in unipolar bacteria. The other observed pattern was named parachute and likely corresponds to a flagellum flipped back and rotating around the cell body; to the best of our knowledge, this was the first time that a flagellum was observed in this position. They identified runs, pauses and reversals in the trajectories for *M*. *magneticum*, (74% had runs, 7% had at least one reversal, and about 19% had at least one pause although some tracks had as many as 5 pauses). Furthermore they observed that during runs, the leading flagellum shows a parachute configuration and the lagging flagellum shows a tuft configuration (see Figs 4 and 5 of ref.^15^). Murat *et al*.^15^ also noted that cell reversals are caused by a change of the rotation direction of both flagella, with the parachute pattern becoming a tuft and the tuft pattern changing to a parachute. They also found instances where the bacterium would tumble in place with both flagella showing a parachute pattern or both showing a tuft pattern, likely corresponding to the pauses during swimming. The results of Murat *et al*. question the commonly held belief that flagella always rotate extended away from the cell, as has been seen in unipolar bacteria. It is usually assumed that bipolar bacteria would either have to coordinate flagella at both poles to rotate in opposite directions; or that it would swim as a unipolar bacterium, with only one flagellum active at a time.

Recent studies on *Shewanella putrefaciens*, a marine and sandstone organism^16^ and *P*. *putida*^17^ using fluorescently labeled flagellin, show that unipolar bacteria are also able to flip their flagella or flagellar bundles. *S*. *putrefaciens* has a primary single polar flagellum and additional lateral flagella responsible for cell realignment. Kuhn *et al*.^16^ used mutants lacking the lateral flagella and labeled the flagellin to study its strategy to escape confined environments. While free-swimming cells displayed a run-reverse-flick strategy, trapped bacteria alternated direction of flagellum rotation but in cases where this effort did not suffice to free the cell the flagellum was observed to flip back to wrap around the body. Interestingly in this case the waveform of the flagellum did not change in relation to the substratum, but the body translated backwards rotating in a screw-like form. By immersing free-swimmers in an environment with increased viscosity (instead of confining them to small regions) they observed the same flagella wrapping but this time the flagellum rotated around the body with no effective body translation. Moreover, the fraction of bacteria displaying a wrapped flagellum increased with viscosity. On the other hand, *P*. *putida* has been reported to display a wrapped flagellar swimming mode in bulk without being trapped or increased viscosity, where Hintsche *et al*.^17^ attributed the transition to be initiated by the flagellar motor reversal. Unlike *S*. *putrefaciens*, *P*. *putida* has a polar flagella tuft instead of a single polar flagellum, indicating that such mode is not exclusive of monotrichous bacteria.

Here we investigate the swimming strategy and coordination of the two flagellar bundles of bipolar lophotrichous bacteria by studying the gram-negative stomach pathogen *H*. *suis*. *H*. *suis* has 4–10 sheathed flagella at each end of its tightly coiled body^18^, instead of a single flagellum at each end as the previously studied bipolar bacteria *M*. *magneticum*. *H*. *suis* colonizes the fundic and pyloric gland zone of the stomach in pigs and is associated with ulceration of the non-glandular stomach^19^. Moreover, it is the second-most common *Helicobacter spp*. in humans suffering from gastric disease, outranked only by *H*. *pylori*. Like *H*. *pylori*, *H*. *suis* also has to get across the protective gastric mucin layer of the stomach, a viscoelastic medium that gels at low pH, in order to establish a colony close to the epithelial cells. In this work we have taken advantage of the large number of flagella at each pole of *H*. *suis* leading to a bundle thick enough to be visualized without a fluorescent label using phase contrast microscopic video imaging at high magnification and fast frame rates (100X, 100–200 fps). At such high magnifications (hence small fields of view) only a few bacteria can be imaged, and the bacteria with visible flagella bundles have to be identified manually by watching the movies. Using the same single cell method of analysis with the program CellTool^20^ as described in our previous work^14^ we simultaneously measured shape, swimming speed and body rotation rate while inspecting the movies to observe the flagellar bundles of *H*. *suis* swimming in *Brucella* broth (BB10) and 15 mg/mL porcine gastric mucin (PGM). We observed that *H*. *suis* can swim with the leading flagellar bundle rotating wrapped around the body while the lagging flagellar bundle is extended behind the cell; it can also have both bundles extended, or both wrapped. Our observations are similar to previous findings of wrapped and extended flagella in the bipolar *M*. *magneticum*^15^, and in the unipolar *Shewanella putrefaciens*^16^ and *P*. *putida*^17^. In the two modes with both bundles extended or both wrapped, the *H*. *suis* bacterium swims at a lower speed and with more trajectory reorientations. In addition, while Murat *et al*.^15^ observed fluorescence patterns that were associated with two likely configurations, our movies capture images of the rotating bundles and the transition event between the two flagella configurations, similar to the data presented by Hintsche *et al*.^17^.

## Results and Discussion

### Part I. Experimental observation of various swimming modes in *H. suis*

We examined the swimming modes of live bacteria by video microscopic tracking in culture broth BB10, as well as in PGM at pH6. The studies in PGM were made at a concentration of 15 mg/ml which corresponds to the concentration of the non-adherent outer layer of mucus and has previously been shown to not exhibit significant non-Newtonian effects at pH6^8,21,22^. In order to visualize flagella we recorded the videos at 100–200 fps with 100X magnification and focused on imaging individual bacteria in the center of the slide, to minimize edge effects^23^.

**Figure 1:**
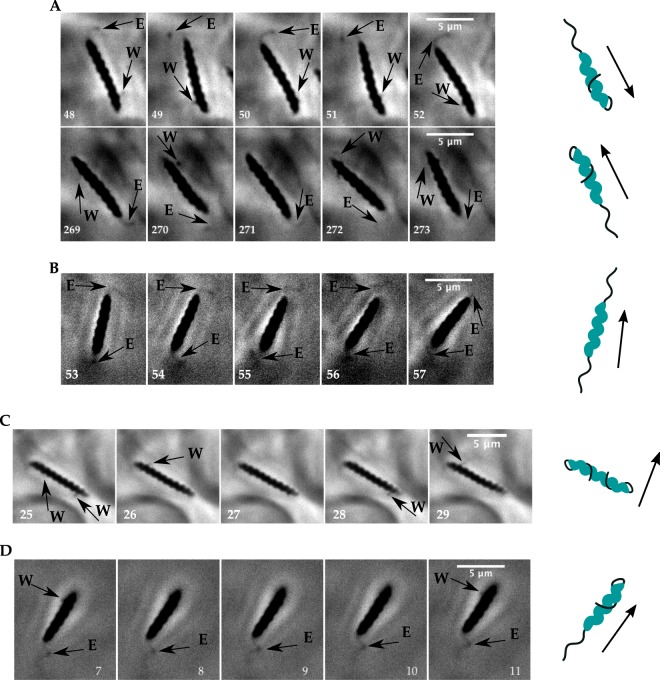
*H*. *suis* flagella dynamics. Arrows point to flagellar bundles, E denotes extended and W denotes wrapped; the cartoons illustrate the flagellar bundle configurations, where flagellar helicity was chosen at random. (**A**) Movie recorded at 100X and 100 fps in PGM pH6. The bacterium swims with the leading flagellar bundle wrapped around body and lagging flagellar bundle in extended mode in frames 48–52 (run# 33 in Supplementary Table S1). To reverse swimming direction the bundles switch modes in frames 269–273 (run# 34 in Supplementary Table S1). (**B**) Movie recorded at 100X and 100 fps in BB10 (run# 12 in Supplementary Table S1). The bacterium swims with both bundles in extended position. (**C**) Movie recorded at 100X and 187 fps in PGM pH6. The bacterium swims with both bundles wrapped around body. (**D**) Movie recorded at 100X and 100 fps in BB10. The bacterium swims with active wrapped bundle and inactive extended bundle.

The movies show that the flagellar bundles of *H*. *suis* can assume two different configurations while swimming, which we named extended and wrapped (Fig. 1). Arrows labeled E point to a bundle extended behind the cell, as normally seen in lophotrichous bacteria such as *H*. *pylori* or in peritrichous bacteria such as *E*. *coli*. Arrows labeled W point to a bundle oriented in the wrapped position: the bundle is seen on both sides of the bacterium, indicating it is wrapped around the body. Similar flagellar orientations have been inferred from fluorescent images on *M*. *magneticum*, which has a single flagellum rather than a bundle^15^. Supplementary Movie S1 supports that the bundle is wrapped around the cell, instead of only flipped back and rotating on the side of the body.

There are three possible combinations of the two bundles positions during swimming: extended/wrapped (EW), wrapped/wrapped (WW) or extended/extended (EE). We observed all three modes in broth but only two (EW and WW) in PGM as shown in the examples on Fig. 1A–C. We also captured transitions between all different modes and transitions where the bacterium reverses the direction of swimming, switching the extended bundle to wrapped and the wrapped bundle to extended. It is important to note that because this is a bipolar bacterium, we cannot determine whether the extended flagella configuration is pushing or pulling the bacterium based only on the direction of swimming and position of bundle. Figure 1D shows consecutive frames of a bacterium swimming with the leading bundle active in wrapped mode while the lagging extended bundle rests. Even though the extended bundle is not rotating, the bacterium is able to translate with swimming orientation parallel to the wrapped bundle. That is a strong indication that the wrapped bundle can generate thrust while actively rotating around the body, hence contributing to the swimming speed. During this event, which lasted 0.17 seconds, the body of the bacterium does not seem to rotate significantly and the body maintains a relatively constant alignment angle (see Supplementary Movie S2).

### Transitions between extended and wrapped modes

We observed one bacterium that was swimming in a micro-channel formed in PGM at pH6, where the bacterium swam back and forth (see Supplementary Movie S3). This facilitated recording the video in a higher frame rate (343 fps) and for a longer period of time (~14 seconds). Only one flagellar bundle is visible in this bacterium, and due to the background we are only able to visualize the transition from extended to wrapped. The movie shows the bacterium swimming with the lagging bundle in extended configuration while the body rotates (the 2D projection of the 3D helical body changes shape when the body rotates, analogous to a travelling sine wave). Right before the wrapping event, the bundle slows rotation before coming to a stop while the body rotation follows the same trend and the bacterium stops translating. During the wrapping event, the flagellar tuft remains bundled. After the wrapping event the bacterium swims in the opposite direction, flagella rotate wrapped around the body and the body reverses direction of rotation. This was also observed previously by Kuhn *et al*.^16^ for a single polar flagellum and could be an indication that the flagellar mode transition is a dynamic event triggered by a change in direction of rotation of the flagellar motors, but a more detailed experimental investigation on *H*. *suis* flagellar dynamics has to be conducted to reach a definite conclusion.

To gain insight on this conformational change Kuhn *et al*.^16^ modeled the flagellum as an elastic rod coupled to the fluid through resistive force theory, revealing that the change could be triggered by an increase in the external torque on the body (due to increased viscosity or a trapped bacterium), which would make the flagellum begin to move sideways. After motor reversal, due to the flagellum being moved sideways it would start to pull on the bacterium forcing the flagellum to wrap around the body. Because the flagellum of *H*. *suis* displays a wrapped mode after a reversal in body rotation (resulting from a switch in motor rotation) in broth as well as PGM at various viscosities, it is likely that the external torque applied on *H*. *suis* body is always above the torque threshold to cause the flagellar instability, independently of viscosity. However to confirm this, a similar model should be implemented for *H*. *suis*, which has a bundle of multiple flagella rather than a single flagellum, or a new model could be implemented using the more precise but more computationally expensive Method of Regularized Stokeslets.

We captured 8 transitions that lasted an average time of 131 ± 47 ms, consistent with the duration of reversals observed in unipolar bacteria^10^. The transition event described here and seen in Supplementary Movie S3 is also seen during flagellar bundle transitions between wrapped and extended in the other movies that captured a transition, such as Supplementary Movie S4. Even though this transition was also observed in broth, we cannot assume that the transition average time and dynamics in the PGM micro-channel represents the transition in bulk PGM or broth^16^ as the constricted geometry of the channel might impose constraints different from the bulk.

In order to characterize the three swimming modes, we analyzed the movies one bacterium at a time, following the method of Constantino *et al*.^14^, using CellTool^20^ to measure speed, body shape and body rotation rate simultaneously. Among the 28 analyzed bacteria swimming in BB10, flagellar modes could not be determined in 14 of them because one or both flagellar bundles were not visible. Among the other half, 4 bacteria changed modes during the swim while the other 10 swam in only one mode. Among the 19 bacteria swimming in PGM, the flagellar mode for 8 of them could not be determined. Among the 11 remaining, 3 changed modes while the other 8 swam in only one mode. For trajectories where the bacterium changed modes, they were segmented manually into runs of the same mode. Some continuous runs in the same mode had to be segmented because the bacterium got out of focus during the movie.

**Figure 2:**
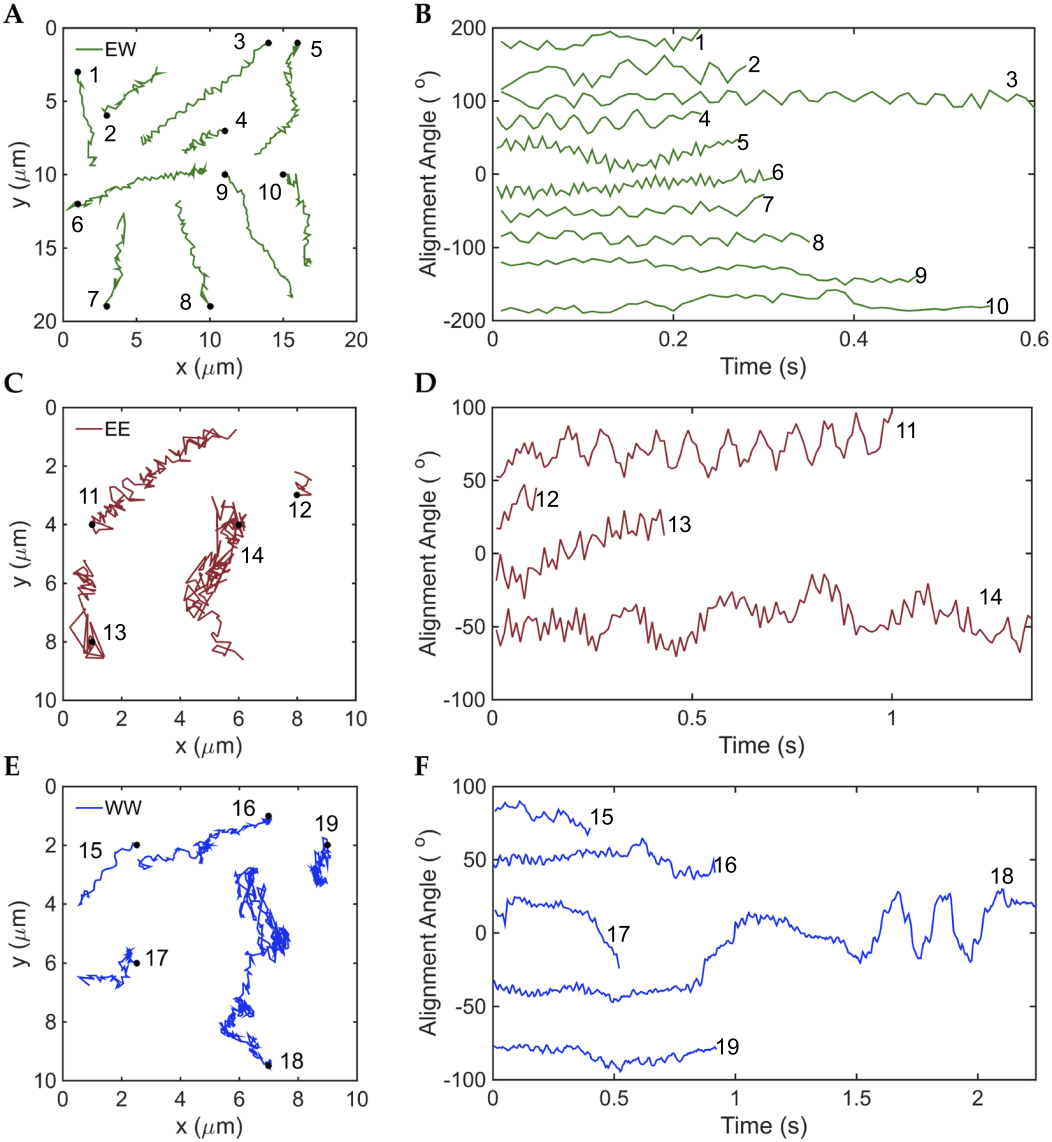
Trajectories and body alignment angle of different swimming modes of *H*. *suis* swimming in BB10. (**A**,**C**,**E**) Trajectories for modes EW, EE and WW respectively. The trajectories were randomly distributed over the figure for better visualization and do not depict the real position on the movie. (**B**,**D**,**F**) Alignment angle of the center-axis of the body with respect to the x-axis of the video for EW, EE and WW modes respectively. The alignment angle values were translated in the y-axis for better visualization.

**Figure 3:**
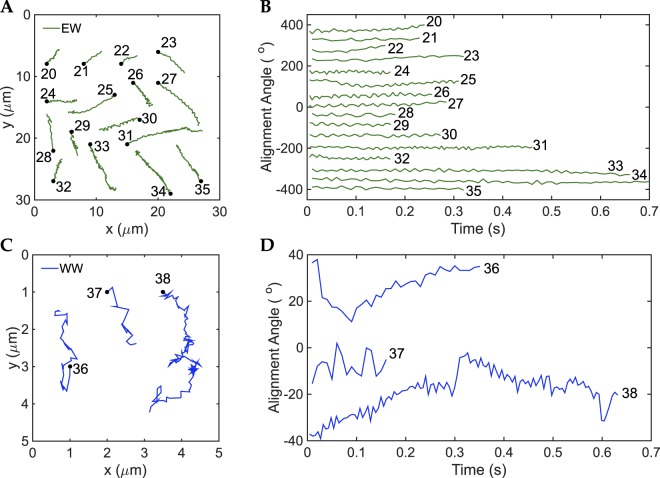
Trajectories and body alignment angle of different swimming modes of *H*. *suis* swimming in 15 mg/mL PGM at pH6. (**A**,**C**) Trajectories for EW and WW modes, respectively. The trajectories and alignment angles were shifted so as to be distributed over the figure for better visualization and do not depict the real position on the movie. (**B**,**D**) Alignment angle of the center axis of the body with respect to the x-axis of the image versus time for EW and WW modes respectively. There were no runs in EE mode observed for PGM in this set of bacteria.

We captured a total of 19 runs in BB10; 10 runs (53%) in EW mode, 4 runs (21%) in EE mode and 5 runs (26%) in WW. In PGM also we captured 19 runs; 16 runs (84%) in EW mode, no runs in EE mode and 3 runs (16%) in WW mode in PGM. Due to the small sample size the numbers are only of qualitative significance. Nevertheless, the EW mode is the most frequently observed one in both media, BB10 and PGM, and moreover it occurs more frequently in the more viscous PGM as compared to BB10. Figures 2 and 3 show the trajectories and the alignment angle defined as the angle between the body axis and the x-axis of the image frame for each run (Constantino *et al*.^14^), sorted by swimming mode in BB10 and PGM respectively. We also measured the size and shape parameters of all the *H*. *suis* bacteria that we imaged. Supplementary Table S1 gives the speed, body rotation rate and cell shape parameters for all of the observed runs in BB10 and PGM. An examination of the shape parameters clearly shows that *H*. *suis* vary in length from 6–10 μm and have 4–10 helical turns, confirming previous findings that *H*. *suis* is longer and more coiled than *H*. *pylori*^18^.

#### Extended/wrapped runs

While swimming in EW mode, *H*. *suis* moves in helical trajectories that can be straight or curved (Figs 2A and 3A), with swimming orientation parallel to the extended bundle. Among the 26 observed occurrences of bacteria swimming as EW, all bacteria swam with the leading flagella bundle in wrapped position and the lagging flagella bundle in extended position. The alignment angle (Figs 2B and 3B) oscillates over time, consistent with the helical trajectories and with previous observations of *H*. *pylori*^14^, where we observed “wiggling” trajectories caused by rotation of flagellar bundles which have a fixed orientation relative to the cell body. Hence, the alignment angle oscillation frequency (inverse of the period of oscillation) provides an estimate of the body rotation rate for EW mode.

#### Wrapped/wrapped and extended/extended modes

Both EE (Fig. 2C) and WW (Figs 2D and 3C) trajectories have more reorientations than the EW trajectories. We observed parallel and perpendicular swimming orientation with respect to the flagella bundles. The alignment angle (Figs 2D,F and 3D) shows slow oscillations over time along with the usual fast frequency component that corresponds to body rotation. The slow oscillations of the alignment angle correlate with the large reorientations observed in the corresponding trajectories (see for example trajectory 14 and 18 in Fig. 2, and 38 in Fig. 3). A careful inspection of the movies shows no correlation between the slow alignment angle oscillations and body rotation; they arise because the overall direction of swimming is changing due to large reorientations. In some instances the bacterium body does not appear to rotate in EE and WW modes (Supplementary Movies S4 and S5), which could be an indication that the bundle in extended mode rotates one way, while the wrapped mode rotates the opposite way. In that case, bundles of a bacterium swimming in WW mode or EE mode (where the two bundles are parallel) will not produce thrust in the same direction, hindering the motion.

**Figure 4:**
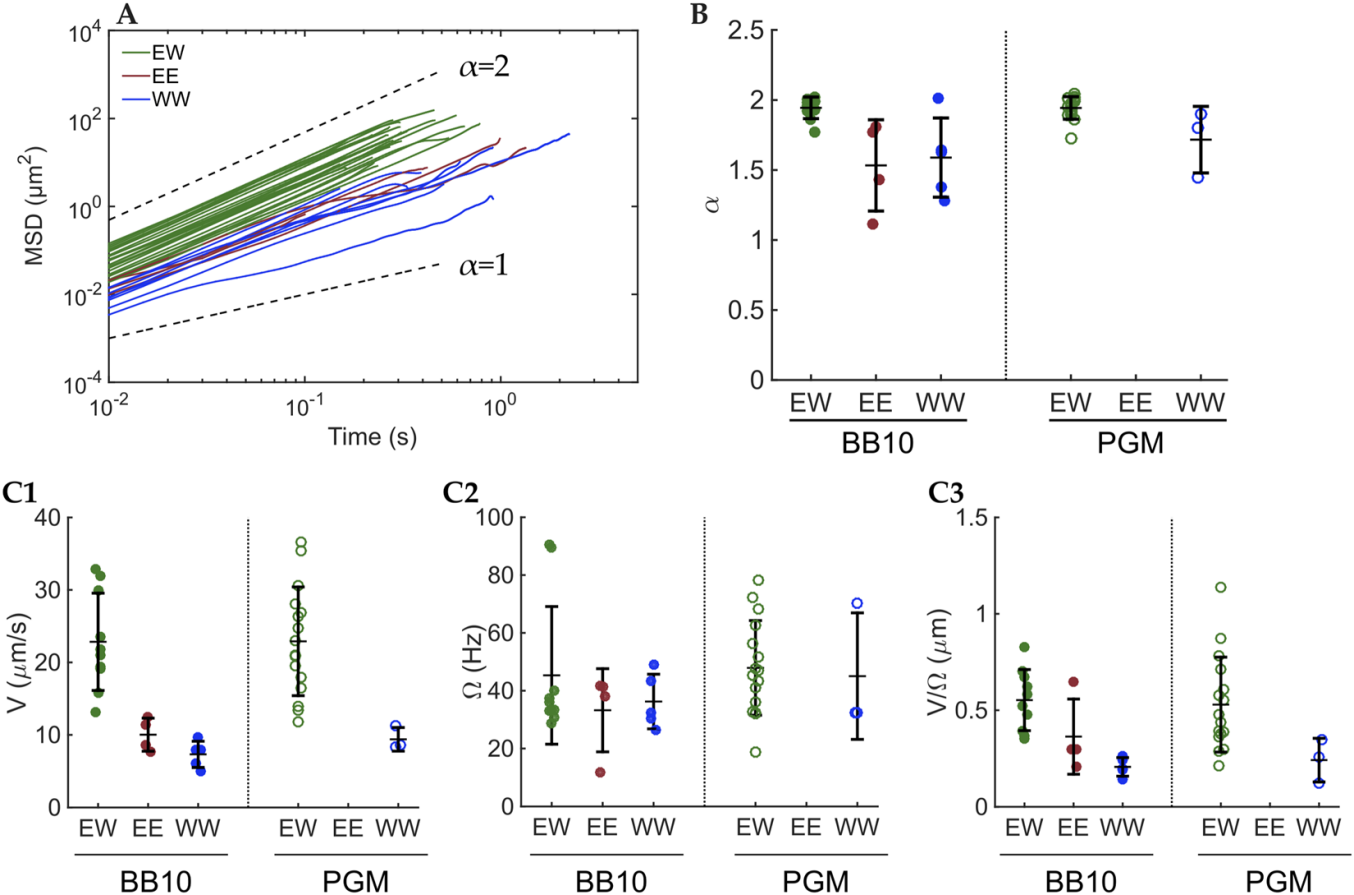
MSD and the exponent α for each mode along with speed and body rotation rates for the different modes. (**A**) MSD vs. time of each trajectory. The green lines are the MSD of EW mode trajectories, maroon lines are the MSD of EE mode trajectories and blue are the MSD of WW mode trajectories. The trajectories in BB10 and PGM were plotted together. Black dashed lines are reference lines for α = 1 and α = 2. (**B**) Dot plot of α-values for each trajectory separated by mode and medium. The central horizontal lines are the mean values and the vertical bars are the standard deviations. (**C**) Speed (V), alignment angle frequency (Ω) and V/Ω for the swimming modes of *H*. *suis* in BB10 and PGM. Green symbols correspond to EW mode, maroon to EE and blue to WW. Closed circles correspond to BB10 and open symbols to PGM. The horizontal lines indicate the mean while the vertical lines are the standard deviation. 1. Speed of each run separated by mode and media. Each point was calculated as an average of the instantaneous speeds during the individual run. 2. Alignment angle frequency separated by mode and media. Each point was calculated as the average of the frequencies for each run. 3. V/Ω separated by mode and media. Each point was calculated as the average speed of one run divided by the average rotation rate of the same run.

Figure 4A shows the mean square displacement (MSD $(t)={ < (\vec{r}(t)-\vec{r}(0))}^{2} >$) for each trajectory in BB10 and PGM. It is clear that bacteria swimming in EW mode travel longer distances than EE and WW. The MSD follows a power law time dependence MSD $(t)=A{t}^{\alpha }$, where $A=2dD$ is a proportionality constant, *d* is the dimension of the trajectories and *D* is the diffusion constant^21,24^. For diffusive particles, $\alpha =1$; for sub-diffusive particles, $\alpha  < 1$; and for super-diffusive particles $\alpha  > 1$. Ballistic motion at constant speed would correspond to $\alpha =2$. Self-propelled particles exhibiting runs and re-orientations, such as flagellated bacteria, will show a super-diffusive behavior. We fitted the first 50% of each MSD to a power law and obtained their individual values of *α*, shown in Fig. 4B and Supplementary Table S1. For EW trajectories in BB10 and PGM, $\alpha  \sim 2$, indicating that bacteria swimming in EW mode exhibit a ballistic motion, with constant speed. On the other hand, EE and WW modes have a smaller *α*, but still super-diffusive, indicating that the motion is self propelled but not ballistic, consistent with the trajectory reorientations.

### Speed and body rotation rate

Figure 4C1 shows the speed (calculated as the average of the instantaneous speeds) of each run for the different swimming modes in BB10 and PGM. There were no observations of EE mode in PGM. Figure 4C1 and Supplementary Table S2 clearly show that regardless of media, EW mode has faster speeds than EE and WW. As our theoretical model shows later, the decreased speeds in EE and WW modes can be interpreted as bundles rotating in the same direction, hence providing thrust in opposite directions.

Figure 4C2 shows the frequency of alignment angle of each run for the different swimming modes in BB10 and PGM, calculated by measuring the period between two consecutive peaks in the alignment angle^14^. For EW mode, we assume that the flagellar bundle in extended mode has a fixed orientation relative to the cell body, so when the body counter-rotates, the body alignment angle with respect to the x-axis oscillates with the same period as the body rotation, similar to *H*. *pylori*. Careful inspection of the movies confirms the alignment angle period of EW mode matches the period of rotation of the body. When swimming in EE or WW mode, the alignment angle method does not capture body rotation rate, the variation in alignment angle arises from the bacterium reorienting itself. Figure 4C2 also shows that the body rotation rate in EW mode increases in PGM, indicating that the flagellar motors rotate faster in PGM. Figure 4C3 shows the parameter V/Ω, a measurement of the travelled speed per body rotation, and it is only meaningful for the EW mode, as discussed above. V/Ω is the same for BB10 and PGM, which is an indication that the travelled distance per rotation does not depend on the medium. This is in agreement with Resistive Force Theory^25^ and Regularized Stokeslet^26,27^ models for low Reynolds number swimmers in Newtonian fluids, which finds that V/Ω depends only on the bacterium geometry.

### Imaging the motion of large populations of bacteria at low magnification

**Figure 5:**
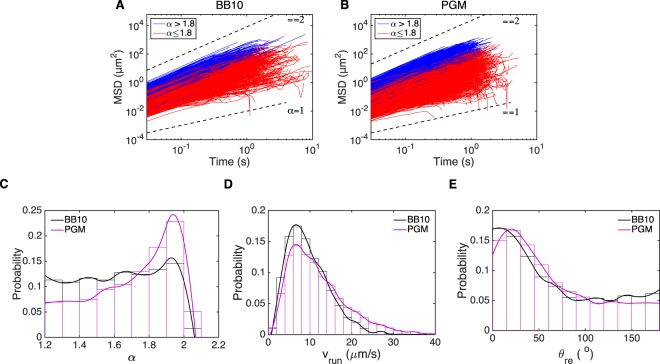
(**A**,**B**) Analysis of MSD vs. time of all trajectories in BB10 (**A**) and PGM (**B**) along with distributions of the exponent α, run speed and reorientation angle. The blue (red) lines are the MSD of trajectories with α > 1.8 (α ≤ 1.8). Black dashed lines are reference lines for α = 1 and α = 2. (**C**) Smooth histogram of the α distribution of trajectories in BB10 (black) and PGM (magenta) (**D**,**E**). Smooth histogram of v_run_ (**D**) and θ_re_ (**E**) of *H*. *suis* swimming in BB10 (black) and PGM (magenta) at pH6. The histograms have 0.1 bin size for α, 2 μm/s bin size for v_run_ and 15° bin size for θ_re_.

We were only able to image 10’s of bacteria with the high magnification, fast frame rate imaging and manual detection of bacteria with visible flagella. To study the swimming behavior of large population of *H*. *suis* bacteria we recorded videos at 40X magnification and 33 fps, which provides a larger field of view to image a larger sample of bacteria and enough time to capture longer trajectories. The movies were tracked with the software PolyParticleTracker^28^ and their trajectories were segmented into runs by identifying reorientation events based on the methods of Theves *et al*.^10^ and Son *et al*.^29^, briefly explained in Methods. After segmentation, we obtain *α* for each run (as explained in the previous section) and exclude tracks with $\alpha  < 1.2$ to eliminate immobile bacteria^22^. In this experiment and analysis we do not look at high-magnification single bacteria movies, hence we do not classify the trajectories by visually determining the flagella configuration. Instead we use the results from the previous section to characterize the different modes using the time dependence of the MSD, MSD $(t)=A{t}^{\alpha }$. An examination of Fig. 4B reveals that in the high magnification, direct flagella visualization experiment the EW mode was characterized by ballistic trajectories with $\alpha  > 1.8$, whereas the EE and WW modes had superdiffusive random walk trajectories with $\alpha \le 1.8$. Hence, we use the exponent *α* to categorize these trajectories into those corresponding to EW modes. We choose the cutoff value as $\alpha =1.8$ to be consistent with the results of Fig. 4B; however a different cutoff value would not change our overall conclusion although it would give different numbers for the fraction of trajectories in the two different categories. Figure 5A,B show the MSD vs time plots for all trajectories recorded in BB10 (1837 trajectories) and in PGM (9067 trajectories) colored according to the two different categories with the cut-off at $\alpha =1.8$. Figure 5C shows that the distribution of *α* is significantly different in BB10 as compared to PGM, with PGM exhibiting a smaller fraction of the superdiffusive random-walk trajectories (61% in PGM vs 77% in BB10) similar to the EE and WW trajectories shown in Fig. 4 and perhaps explains our observation that we did not capture any EE mode in the high magnification flagella imaging experiment in PGM (see Fig. 4B).

To further characterize the motility of the population swimming in broth and PGM, we analyzed the mean speed during a run (v_run_), calculated as the average of the instantaneous speed during the run; and the turn angle after a reorientation event (θ_re_). See Methods for a description of how the trajectories were segmented into runs and reorientation/turns). Figure 5D,E show the histogram of these quantities for over thousands of trajectories of bacteria for both media. Only very small differences are observed in v_run_ and θ_re_ between BB10 and PGM; the mean ± standard deviation of v_run_ increases slightly from 10 ± 5 μm/s in BB10 to 12 ± 7 μm/s in PGM.

However there is a higher probability of observing faster v_run_ in PGM is higher at faster speeds; e.g. 31% of bacterial run speeds are higher than 14 μm/s in PGM as compared to 19% in BB10. Our results suggest that *H*. *suis* swims faster in 15 mg/mL PGM at pH6 than in broth; a similar result was found for *H*. *pylori*^8^.

The distribution of reorientation angles in BB10 shows a narrow peak at low angles accompanied by a broad distribution at high turn angles. The low angle peak characteristic of a small change in the direction of motion before and after reorientation event probably arises when the bacterium changes from EW mode to EE or WW, or when there is a speed change in the trajectory, or when the bacterium moves in and out of focus. The broad distribution at large angles is indicative of reversal events with turn angles ranging around 105–180°, where the bacterium still swims in EW mode but switches the extended flagella to wrapped and *vice-versa*. A similar reorientation angle distribution has been seen in the unipolar soil bacteria *P*. *putida*^11^. The preferred turning angle in PGM is slightly increased as compared to broth, while the percentage of reversals (θ_re_ > 105°) decreases slightly from 28% in BB10 to 23%. This result agrees with previous observations that the percentage of reversals decreases in *H*. *pylori* swimming in PGM relative to BB10^8^.

### Part 2. Theoretical model for propulsion by bipolar flagellar bundles

**Figure 6:**
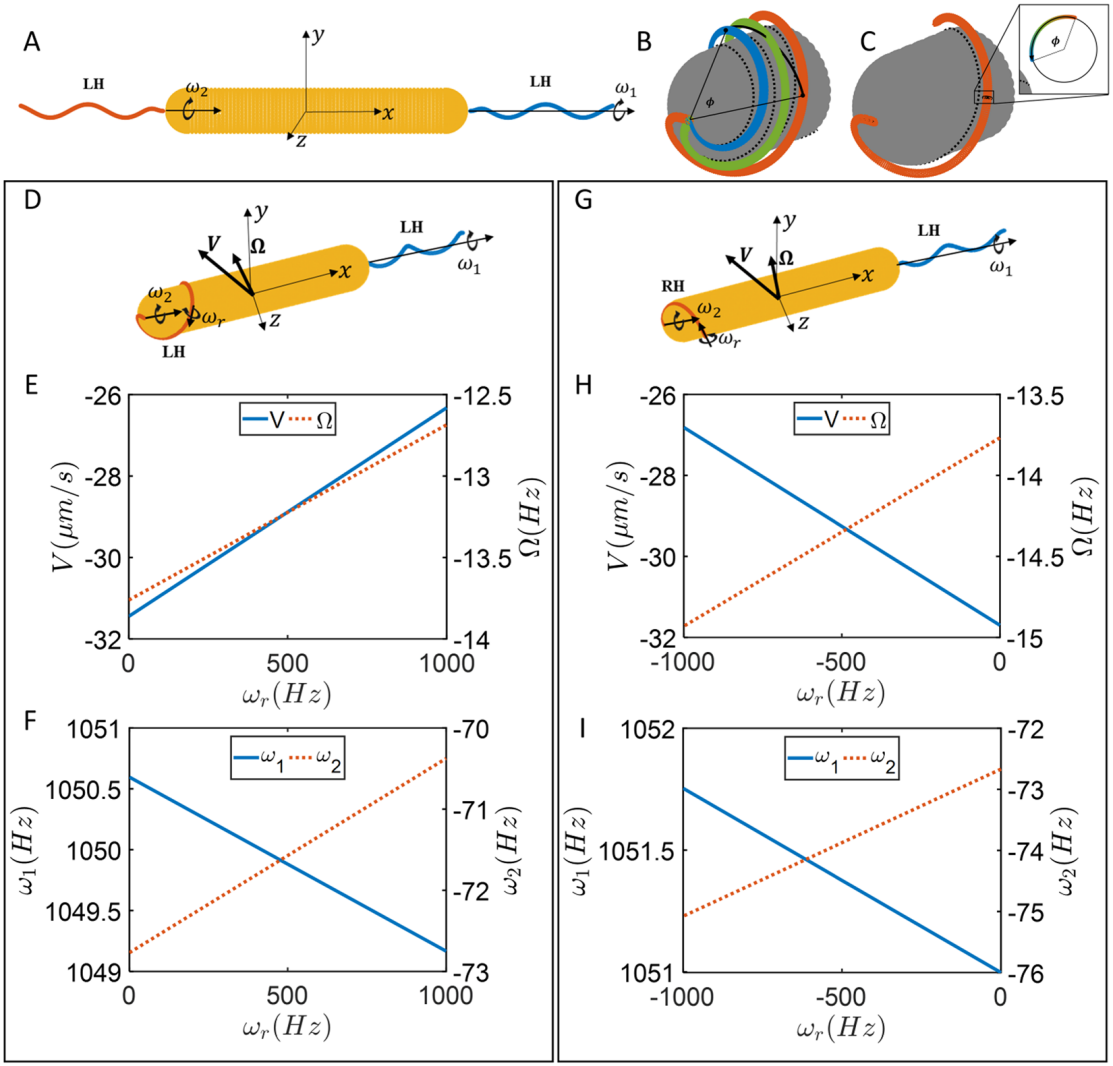
(**A**) Model for extended-extended mode of motion. Both bundles are left-handed and the motor torque of *2000 pN nm* is applied in positive x-directions for each bundle. (**B**) Rigid body rotation of the flagellum in wrapped mode. (**C**) Rolling rotation of the filament representing the bundle in wrapped mode; the filament maintains its position relative to the cell body and material points on a cross section move around the cross section. (**D**) Model showing extended-wrapped swimming with both bundles left-handed while both motors rotate counterclockwise with same torque, set at 2000 pN nm here. (**E**) Swimming speed and body rotation rate as a function of undetermined rolling rotation rate of wrapped bundle filament. (**F**) Rigid-body rotation rates of each bundle as a function of undetermined rolling rotation rate. (**G**) Model showing extended-wrapped swimming with wrapped bundles right-handed while both motors rotate counterclockwise with same torques as in D. (**H**) Swimming speed and body rotation rate as a function of undetermined rolling rotation rate of wrapped bundle. (**I**) Rigid-body rotation rates of each bundle as a function of undetermined rolling rotation rate.

Here we aim to create a model for propulsion by bipolar flagellar bundles that explains the basic observed characteristics of the different swimming modes of *H*. *suis*. From observations, we know that the modes correspond to the two bundles being extended (E) or wrapped around the cell body (W) in various combinations (EE, EW, and WW). However, the level of detail in our visualization leaves many unknowns. For example, we could not image the precise shape of an extended flagellum, although it may be guessed from observations of other species or SEM images. The precise shape of a wrapped flagellum is even less constrained, and both its shape and position (closeness) to the cell body are unknown. In our model, we try to capture qualitative features of the swimming behavior by making broadly generic assumptions about flagellar geometry and actuation. The particular observed features that we address are: (1) EW swimming has a much higher speed than EE or WW swimming (Fig. 4C1). (2) EW trajectories have a ballistic character, while EE and WW swimming is super-diffusive (and sub-ballistic) (Fig. 4B). Typically, flagellar motors operate in a constant-torque regime of their rotation rate-torque curve^30^. In the results presented below, we assume that both bundles are actuated by flagellar motors turning with constant total torque of 2000 pN nm per bundle^31^, and the direction of rotation is counterclockwise (CCW) for both bundles as viewed from outside the cell body (Fig. 6A). The precise value of motor torque was chosen to approximately match swimming velocities and rotation rates with our experimental observations, but does not affect our theoretical conclusions about *relative* speeds or rotation rates between EE, EW, and WW modes. We assume representative geometries as a single effective helical filament for both the extended (Left handed) and wrapped bundle (detailed in next section). For the wrapped bundle, we investigate the effect of different pitch and handedness of its helical structure, as well as the gap between the wrapped bundle and cell body, since they are not determined from experiments. We assume a rod-like (Table 1), rather than helical cell body shape. In the SI, we show that the choices of gap and rod-like shape do not qualitatively affect our results.

**Table 1:** Rod-shaped cell body dimensions for different swimming modes.

	EE	EW	WW
Length, X_B_ (µm)	6.1	6.3	6.4
Diameter D (µm)	0.8	0.9	0.8

One key difference between the rotational kinematics of a typical extended helical bundle and the wrapped bundle is that the wrapped filament could have two different types of rotations around the cell body. One type is rotation as a rigid body along its axial centerline (Fig. 6B), in which the filament stays in the same geometry without deforming as it moves around the body. The material points of the filament closest to the cell body remain closest to the cell body throughout the rotation. In the other type of rotation the filament “rolls” (Fig. 6C), i.e., it maintains its position relative to the cell body, but at each cross-section of the filament, material points on the surface of the flagellum continually move around the cross section. Both types of rotation are consistent with motor rotation at the base of the filament, and the motor rotation rate is equal to the sum of both rotations. Physically, the rolling degree of freedom should be transient since it requires torsional winding of the flagellum that is eventually opposed by the torsional stiffness of the filament; once the torsional torque from stiffness balances viscous torques (including arising from interactions with the cell body), the rolling type of rotation ceases and the bundle should display only rotation as a rigid filament around its axial centerline. In our results, we do not assume that the rolling rotation has stopped; rather we allow arbitrary prescribed amounts of each type of rotation to occur as long as they are consistent with the total motor torque.

### Numerical methods

We employ the Method of Regularized Stokeslets^26,27^, which is briefly described in the SI and which we previously implemented as described in^14,32,33^, to numerically analyze the swimming dynamics of *H*. *suis* in different modes. The surface of the flagellar filament and the rod-shaped cell body are discretized by 2850 and 2800 points, respectively. We prescribe the relative rotation rate of the flagellar filament with respect to the cell body for different modes of rotation rates separately and calculate instantaneous translational and rotational velocities as well as torques and forces on flagellar filaments^32^. We take advantage of linearity of the viscous flow and symmetry for the flagella to find velocities and forces in the body frame by combining results for separate solutions of each flagellar filament. The symmetry allows us to calculate average quantities by averaging the instantaneous solutions over 20 flagellum rotations of each filament separately while the other filament does not rotate.

#### Flagellar filaments configuration and dimensions

We assume that the centerline of the tapered helices^32,33^ for different modes are along the x-axis. The equations of the centerlines of the filaments are described by

1 $${{\boldsymbol{r}}}_{1,2}(x)=(x\pm \frac{{X}_{B}}{2})\hat{{\boldsymbol{x}}}+(1-{e}^{-{(\frac{2\pi (x\pm {X}_{B}/2)}{{P}_{f}})}^{2}})R(\cos (\chi \frac{2\pi }{{P}_{f}}(x\pm \frac{{X}_{B}}{2}))\hat{{\boldsymbol{y}}}+\,\sin (\chi \frac{2\pi }{{P}_{f}}(x\pm \frac{{X}_{B}}{2}))\hat{{\boldsymbol{z}}})\,$$

where ${P}_{f}\,$is the helical pitch, *R* is the helical radius of the filament, ${X}_{B}$ is the length of the cell body, and χ = ±1 defines the handedness of the filament. The different signs in the equation stand for flagellar filaments at each end. The flagellar filament parameters are given in Table 2. The helical radius for extended and wrapped filaments are defined as,

2 $$R=\{\begin{array}{c}{R}_{f}\\ (D+{d}_{f})/2+{\rm{\Delta }}\end{array}\begin{array}{c}:\,{\rm{e}}{\rm{x}}{\rm{t}}{\rm{e}}{\rm{n}}{\rm{d}}{\rm{e}}{\rm{d}}\,{\rm{f}}{\rm{l}}{\rm{a}}{\rm{g}}{\rm{e}}{\rm{l}}{\rm{l}}{\rm{u}}{\rm{m}}\\ :\,{\rm{w}}{\rm{r}}{\rm{a}}{\rm{p}}{\rm{p}}{\rm{e}}{\rm{d}}\,{\rm{f}}{\rm{l}}{\rm{a}}{\rm{g}}{\rm{e}}{\rm{l}}{\rm{l}}{\rm{u}}{\rm{m}}\end{array}$$

where *D* is the diameter of the cell body, *d*_*f*_ is the diameter of the flagellar filament, and Δ is a gap distance that specifies distance between the surface of the cell body and surface of the flagellar filament. We use ${\rm{\Delta }}={d}_{f}$ for our simulations. In SI, we explore dependence of the results on Δ and find that it does not change swimming velocity by much, but can have up to 50% change in cell body rotation rates. Likewise, in the absence of measurements of the pitch of the wrapped flagellum, here we present results for the case that the wrapped and extended flagella have the same pitch. We explore the effects of varying the pitch of the wrapped flagellum in SI and find that it does not affect qualitative results, and at most 10% changes in rotation rates and velocities.

**Table 2:** Flagellar filament parameters.

Arc length L_arc_ (µm)	Filament diameter d_f_ (µm)	Helical pitch P_f_ (µm)	Helical radius R_f_ (µm)
3.4	0.07	1.58	0.14

### EE Mode

For the extended-extended swimming mode, bundles are assumed to be left handed (LH) at each polar positions as shown in Fig. 6A. Prescribing rotation rates ${\omega }_{1}$ and ${\omega }_{2}$ in the Method of Regularized Stokeslets, the motion of the EE swimmer is described by,

3 $$\{\begin{array}{ccc}V & =+0.0254\,{\omega }_{1} & +0.0254\,{\omega }_{2}\\ {\rm{\Omega }} & =-0.0180\,{\omega }_{1} & -0.0180\,{\omega }_{2}\\ {T}_{1} & =+1.9263\,{\omega }_{1} & -0.0377\,{\omega }_{2}\\ {T}_{2} & =\,-0.0377\,{\omega }_{1} & +1.9263\,{\omega }_{2}\end{array}$$

where *V* and Ω are average translational and rotational velocities of the cell body, ${T}_{1}$ and ${T}_{2}$ are motor torques, and $\,{\omega }_{1}$ and $\,{\omega }_{2}$ are rotations rates of flagella. Units in these equations are $\mu m/s$ for the translational velocity, $Hz$ for the rotational velocities, and $pN\,nm$ for the torques.

If both left-handed bundles rotate CCW (${T}_{1}=2000\,pN\,nm,\,\,{T}_{2}=-\,2000\,pN\,nm$), the average translational and rotational velocities are zero because of symmetry, implying zero displacement for the organism and pure diffusive behavior ($\alpha =1$). However, experiments indicate smaller $\alpha$ and super-diffusive behavior at small time for EE modes which could be explained when one bundle is off-axis and it is not symmetric. Therefore, we examined a case in which one flagellum is slightly off-axis by 10^◦^ from x-direction and rotating along its helical centerline with constant motor torque. Our results show small average translational and rotational velocities of $3.25\,(\mu m/s)$ and $0.2\,(Hz)$ in a direction which is nearly perpendicular to the cell body axis. Such small velocities in a perpendicular direction are consistent with observations, and can combine with random reorientation from thermal noise or bundle configuration and orientation changes to lead to non-ballistic but super-diffusive (1 < α < 2) exponents for MSD in the time scales of observations.

### EW Mode

For the extended-wrapped swimming mode, the extended bundle is left-handed as usual, but since we do not know the handedness of the wrapped bundle, we investigate two different cases, one in which the wrapped bundle is left handed, and the other in which it is right-handed. In addition, the wrapped flagellum could rotate along x-axis or roll around its centerline. This rolling motion gives additional degree of freedom in specifying the rolling motion (${\omega }_{r}$) as shown in Fig. 6, and the total motor rotation rate is $\,{\omega }_{2}+{\omega }_{r}$.

#### EW with left-handed wrapped bundle

Prescribing rotation rates (${\omega }_{1},{\omega }_{2},{\omega }_{r}$) in the Method of Regularized Stokeslets, the motion of the EW swimmer with a left-handed wrapped bundle and left-handed extended bundle (Fig. 6D) is described by

4 $$\{\begin{array}{cccc}V & =\,-0.0275\,{\omega }_{1} & -0.0351\,{\omega }_{2} & +0.0050\,{\omega }_{r}\\ {\rm{\Omega }} & =-0.0174\,{\omega }_{1} & -0.0621\,{\omega }_{2} & +0.0012\,{\omega }_{r}\\ {T}_{1} & =+1.8953\,{\omega }_{1} & -0.1210\,{\omega }_{2} & +0.0030\,{\omega }_{r}\\ {T}_{2} & =-0.1204\,{\omega }_{1} & +25.746\,{\omega }_{2} & -0.0618\,{\omega }_{r}\end{array}$$

Assuming constant CCW motor torques of 2000 pN nm for each of flagellar motors the average swimming velocity, cell body rotation rates, and relative flagellum rotation rates are plotted as a function of rolling rotation rate in Fig. 6E,F.

#### EW with right-handed wrapped bundle

Prescribing rotation rates (${\omega }_{1},{\omega }_{2},{\omega }_{r}$) in the Method of Regularized Stokeslets, the motion of the EW swimmer with a right-handed wrapped bundle (Fig. 5G) is described by

5 $$\{\begin{array}{cccc}V & =\,-0.0275\,{\omega }_{1} & +0.0385\,{\omega }_{2} & -0.0050\,{\omega }_{r}\\ {\rm{\Omega }} & =\,-0.0174\,{\omega }_{1} & -0.0622\,{\omega }_{2} & +0.0013\,{\omega }_{r}\\ {T}_{1} & =+1.8953\,{\omega }_{1} & -0.1107\,{\omega }_{2} & +0.0017\,{\omega }_{r}\\ {T}_{2} & =-0.1204\,{\omega }_{1} & +25.781\,{\omega }_{2} & -0.0622\,{\omega }_{r}\end{array}$$

Considering motor torques with magnitude of 2000 *pN nm* but different directions, the average swimming velocity and rotational rates are plotted in Fig. 6H,I.

#### Summary of EW mode motility

For both left-handed and right-handed wrapped bundles, the variations in velocities ($V,{\rm{\Omega }},{\omega }_{1},{\omega }_{2}$) caused by variations in the unknown rolling rotation rate (${\omega }_{r}$) are small. For example, *V* only varies by 15% throughout the range of possible ${\omega }_{r}$, and ${\omega }_{2}\ll {\omega }_{1}$. Therefore, the propulsion of the EW mode is dominated by the contributions of the extended flagellum. The calculated translational velocities are qualitatively consistent with observed velocities (~29 and ~23 um/s, respectively), and much larger than velocities calculated in the EE and WW (next section) cases. These velocities can explain the ballistic motion observed in the EW mode. Finally, the calculated rotation rate ${\omega }_{1}$ is large, but not extremely so compared to that observed for marine bacteria.

### WW mode

**Figure 7:**
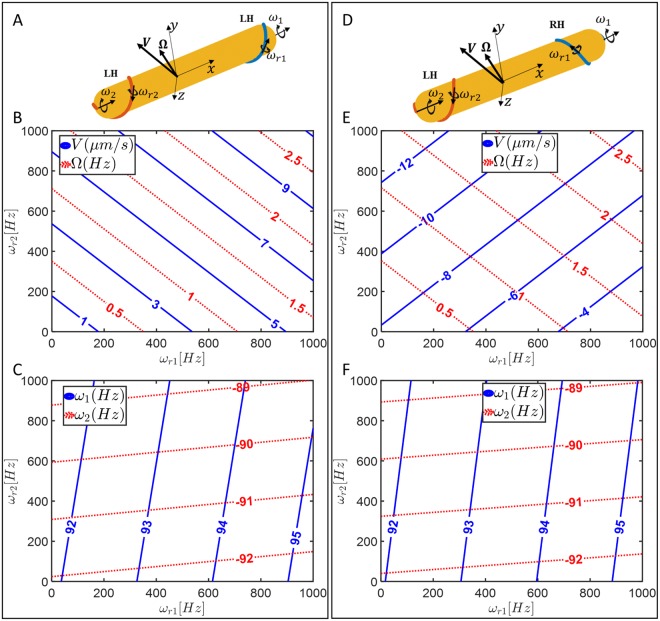
(**A**) Model of bacterium showing wrapped-wrapped swimming with both bundles left-handed while both motors rotate CCW with the same torque, set at 2000 pN nm. (**B**) Swimming speed and body rotation rate as a function of undetermined rolling rotation rates of each bundle. (**C**) Rigid-body rotation rates of each bundle as a function of undetermined rolling rotation rates. (**D**) Model of wrapped-wrapped swimming with one bundle left-handed and the other bundle right-handed while both motors rotate CCW with same torque set at 2000 pN nm. (**E**) Swimming speed and body rotation rate as a function of undetermined rolling rotation rates of each bundle. (**F**) Rigid-body rotation rates of each bundle as a function of undetermined rolling rotation rates.

When both bundles of flagella are left-handed and wrapped around the cell body, there are additional degrees of freedom parameters in the model corresponding to rolling rotations of each bundle, see Fig. 7A. Like the EW mode, we do not know the handedness of the wrapped bundle, so we examine two scenarios, where the bundles have either the same or opposite handedness.

#### WW with two bundles of the same handedness

Prescribing rotation rates (${\omega }_{1},{\omega }_{2},{\omega }_{r1},{\omega }_{r2}$) in the Method of Regularized Stokeslets, the motion of the WW swimmer with two left-handed wrapped bundles (Fig. 7A) is described by

6 $$\{\begin{array}{ccccc}V & =\,-0.0406\,{\omega }_{1} & -0.0406\,{\omega }_{2} & +0.0057\,{\omega }_{r1} & +0.\,0057\,{\omega }_{r2}\\ {\rm{\Omega }} & =\,-0.0698\,{\omega }_{1}\, & -0.0698\,{\omega }_{2} & +0.0016\,\,{\omega }_{r1} & +0.0016\,\,{\omega }_{r2}\\ {T}_{1} & =+21.337\,{\omega }_{1} & -0.4313\,{\omega }_{2} & -0.0740\,{\omega }_{r1} & +0.0108\,{\omega }_{r2}\\ {T}_{2} & =-0.4312\,{\omega }_{1} & +21.289\,{\omega }_{2} & +0.0108\,\,{\omega }_{r1} & -0.0750\,{\omega }_{r2}\end{array}$$

Assuming constant CCW torques for the flagellar motors, the average swimming velocity and rotation rates of the cell body and relative rotation rates are plotted in Fig. 7B,C as contour plots as $\,{\omega }_{r1},{\omega }_{r2}$ are varied.

#### WW with bundles of opposite handedness

Prescribing rotation rates (${\omega }_{1},{\omega }_{2},{\omega }_{r1},{\omega }_{r2}$) in the Method of Regularized Stokeslets, the motion of the WW swimmer with wrapped bundles of different handedness (Fig. 7D) is described by

7 $$\{\begin{array}{ccccc}V & =\,-0.0425\,{\omega }_{1} & +0.0424\,{\omega }_{2} & -0.0057\,{\omega }_{r1} & -0.0058\,{\omega }_{r2}\\ {\rm{\Omega }} & =-\,0.0699\,{\omega }_{1} & -0.0699\,{\omega }_{2} & +0.0016\,\,{\omega }_{r1} & +0.0016\,{\omega }_{r2}\\ {T}_{1} & =+21.337\,{\omega }_{1} & -0.4174\,{\omega }_{2} & -0.0738\,{\omega }_{r1} & +0.0088\,{\omega }_{r2}\\ {T}_{2} & =-0.4165\,{\omega }_{1} & +21.433\,{\omega }_{2} & +0.0086\,\,{\omega }_{r1} & -0.0746\,{\omega }_{r2}\end{array}$$

Assuming constant CCW torques for the flagellar motors, the average swimming velocity and rotation rates of the cell body and relative rotation rates are plotted in Fig. 7E,F.

#### Summary of WW mode motility

For either combination of wrapped bundle handednesses, like the EW mode, the changes in rolling rotations ${\omega }_{r1}$ and ${\omega }_{r2}$ do not cause large variations in ${\omega }_{1}$ and ${\omega }_{2}$ which are both close to 90 ± 5 *Hz* for motor torques 2000 $pN\,nm$. Like the EE mode, swimming velocities are small since the two bundles tend to cancel each other’s propulsion. Such small velocities are consistent with observations, and can combine with random reorientation from thermal noise or flagellar bundle configuration changes to lead to non-ballistic but superdiffusive (α < 2) MSD exponents in the time scales of observations.

## Conclusions

We were able to visualize the thick flagellar bundles of the bipolar bacterium *H*. *suis* by using phase contrast microscopy and simultaneously tracking its motion. Our study shows that, regardless of media, the flagellar bundles of *H*. *suis* can assume one of two configurations interchangeably: extended away from the body, such as a normal pusher/puller bacteria, corresponding to the tuft pattern observed in *M*. *magneticum* by Murat *et al*.^15^; and wrapped, where the flagella bundle almost reverses its orientation to be close to the body and rotates wrapped around the body, corresponding to the parachute pattern observed by Murat *et al*.^15^. *H*. *suis* predominantly swims with the lagging flagella extended behind the body and the leading flagella wrapped around the body (EW mode). During a smaller fraction of the runs, *H*. *suis* was observed to swim with both bundles extended away from the body (EE mode) or wrapped around the body (WW mode), however when swimming in these modes the bacterium speed is much reduced and the trajectories have many more reorientations, suggesting that in the EE case the two bundles negate each other’s action. The trajectories in the EW mode are almost linear, whereas in the WW and EE modes the bacteria travel lesser distances and display trajectories that show characteristics in between a ballistic and a random-walk motion. The EW mode occurs more frequently than the other two (EE or WW) irrespective of medium. Moreover it occurs more frequently in the more viscous PGM as compared to BB10, although the reason for this observation is not clear and needs further investigation. Similar observation of increased EW mode with increasing viscosity was also noted for *S*. *putrefaciens* by Kuhn *et al*.^16^. The characteristics of faster EW swimming and slower EE and WW swimming are qualitatively explained by our theoretical model. Even though the details of the wrapped configuration and kinematics are not known, the model suggests that swimming properties are dominated by the extended flagellar bundle.

## Methods

### *H. suis* culturing

*H*. *suis* bacteria strain HS5 were grown from frozen aliquots on fresh biphasic *Brucella* agar plates supplemented with 20% fetal bovine serum, Vitox and Skirrow supplements and HCl to a pH of 5 under microaerobic conditions (80% N_2_, 10% CO_2_, 10% O_2_; 37 °C, 48–72 hours, on shaker), as described previously^18^. They were passaged to new plates once more before the experiments.

### Bacterial suspension in different media

PGM stock solution was prepared to a final concentration of 15 mg/ml as described previously^14^. PGM solutions were incubated at 37 °C with 10% 0.1 M phosphate/succinate buffer (at pH6) 45 minutes before adding 10% of bacteria liquid culture. The BB10 samples were prepared similarly, with and without the addition of buffer. The final samples were incubated for 45 minutes to 2 hours before use.

### Microscopy and imaging

Microscope slides were prepared as described previously^14^. The bacteria were imaged with an Olympus IX70 inverted microscope at room temperature. Videos were recorded with Zyla 5.5 sCMOS Andor camera (6.5 μm per pixel), controlled with software Micromanager. The measurements for the rotation analysis were obtained with a 100X phase contrast oil-immersion lens (1.25 NA) and recorded at 100 fps or higher frame rate (depending on the selected region of interest). The measurements for the population speed analysis were obtained with a 40X phase contrast lens (0.65 NA) and recorded at 33 fps.

### High magnification and fast frame rate trajectory analysis

The trajectories obtained from the digitized movies were analyzed to obtain cell shape, swimming speed, rotational rate and reorientations using CellTool^20^ based methods identical to those described in^14^. The different flagella configurations, extended vs wrapped were identified by visual inspection of the movies. This was a very labor intensive method.

We note, that recording movies at high magnification and fast frame rates reduces the field of view, thereby reducing the number of bacteria imaged and due to reduced depth of focus it reduces the amount of time a bacterium remains in focus. Because *H*. *suis* is a long bacterium, sometimes part of its body gets out of focus causing the apparent body length to change between frames which adds an error to the tracked position. Furthermore limitations on the size of the movie lead to shorter total duration as many more data frames are recorded. The alternative method based on fluorescent imaging of GFP or other fluorophore labeled flagellin makes it easier to obtain high quality images to elucidate the details of flagella motion; however these labeling methods are perturbative and make it difficult to simultaneously image cell body and flagella rotation. Thus phase contrast and fluorescent imaging of flagella may be viewed as complementary methods.

### Trajectory segmentation method for 40X movies at 33 fps

**Figure 8:**
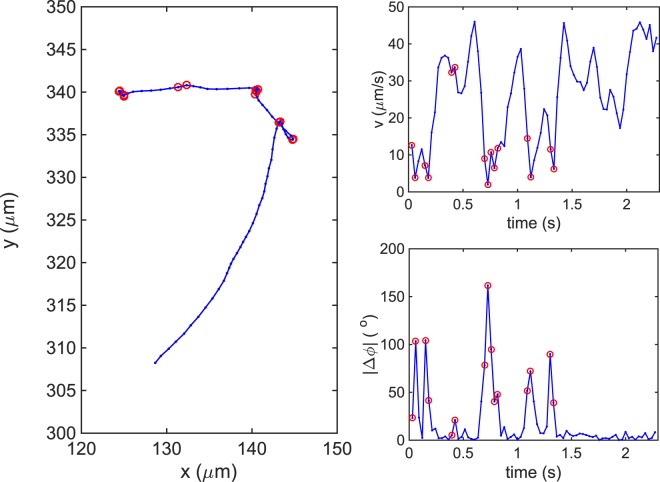
Trajectory of *H*. *suis* swimming in BB10 (**A**) and its respective instantaneous swimming speed (**B**) and absolute angle change, |Δϕ|, over time (**C**). The red circles indicate reorientation events.

The trajectories were segmented into runs based on the methods of Theves *et al*.^10^ and Son *et al*.^29^. Figure 8 shows a typical trajectory of *H*. *suis* swimming in liquid media, along with its instantaneous speed *v* and absolute angle change over time $|{\rm{\Delta }}\varphi |$. The red circles indicate reorientation events, located by looking for large changes in the maximum of $|{\rm{\Delta }}\varphi |$ and/or the minimum of *v* according to Eqs 8 and 9:

8 $$|{\rm{\Delta }}\varphi ({t}_{max})| > \gamma \sqrt{2\,{D}_{{\rm{rot}}}\tau },$$

9 $$Max(|v({t}_{min}+\tau )-v({t}_{min})|,|v({t}_{min}-\tau )-v({t}_{min})|) > \beta v({t}_{min}),$$

where ${t}_{max}$ is the time at which $|{\rm{\Delta }}\varphi |$ is maximum; ${t}_{min}$ is the time at which *v* is minimum; $\tau =0.03\,$s is the time between frames; *γ* is a threshold variable that determines how much larger than the rotational diffusion constant $\,{D}_{{\rm{rot}}}$ that $|{\rm{\Delta }}\varphi \,({t}_{max})|$ has to be; and *β* is a threshold variable that determines how much larger than $v({t}_{min})$ that the speed change has to be to be considered a reorientation event. We found that $\gamma =15$ and $\beta =2$ were enough to capture the reorientation events. The rotational diffusion constant $\,{D}_{{\rm{rot}}}$ for *H*. *suis* was estimated as that of an ellipsoid with semi-minor axis *a* = 0.5 μm and semi-major axis $b=6$ μm at room temperature T = 298 K,

10 $$\,{D}_{{\rm{rot}}}=\frac{{k}_{{\rm{B}}}T(\mathrm{ln}(\frac{2b}{a})-0.5)}{8\pi \eta {b}^{3}/3}\approx 0.0072\,{{\rm{rad}}}^{{\rm{2}}}/{\rm{S}}$$

where ${k}_{{\rm{B}}}$ is the Boltzmann constant and $\eta$ is the viscosity of the medium.

The bacterium is assumed to stay in reorientation event while the local angle changes was larger than $|{\rm{\Delta }}\varphi ({t}_{{\rm{\max }}})|$ and if the displacement of the bacterium was smaller than that of Brownian motion:

11 $$||{\rm{\Delta }}\varphi ({t}_{{\rm{\max }}})|-|{\rm{\Delta }}\varphi ({t}_{{\rm{\max }}}+{\rm{\Delta }}t)|| > {\rm{\Gamma }}|{\rm{\Delta }}\varphi ({t}_{{\rm{\max }}})|,$$

12 $${({\boldsymbol{r}}({t}_{{\rm{\min }}}+{\rm{\Delta }}t)-{\boldsymbol{r}}({t}_{{\rm{\min }}}))}^{2} < 4D{\rm{\Delta }}t,$$

where $D={k}_{{\rm{B}}}T\frac{\mathrm{ln}(2b/a)}{6\pi \eta b}$ is the translational diffusion of an ellipsoid moving at random^34^ and ${\rm{\Gamma }}$ is the percentage change in angle for which the bacterium is still considered to be in reorientation event. We found that ${\rm{\Gamma }}=0.9$ made the best identification. For *H*. *suis* $D=0.12$ μm^2^/s.

The trajectories were segmented according to reorientation events.

All bacterial cultures and imaging studies reported here on *H*. *suis* in purified porcine gastric mucin and broth were carried out under appropriate institutional approvals in Biosafety Level 2 laboratories. Specific protocol approval numbers are: Boston University IBC (institutional Biosafety Committee) approval number 17–1968 (P.I., R. Bansil); Ghent University Biosafety approval number *T_72_0013*, *activity number 2 (*P.I., F. Haesebrouck).

## Electronic supplementary material

Supplementary Information File

Supplementary Movie S1

Supplementary Movie S2

Supplementary Movie S3

Supplementary Movie S4

Supplementary Movie S5

## Data Availability

All relevant data generated or analyzed during this study are included in this published article (and its Supplementary Information files). Additional information is available from the corresponding author on reasonable request.
