# Supplementary material for: Bipolar lophotrichous Helicobacter suis combine extended and wrapped flagella bundles to exhibit multiple modes of motility
Source: Sci Rep. 2018 Sep 26;8:14415. doi: 10.1038/s41598-018-32686-7 (PMC6158295; doi:10.1038/s41598-018-32686-7)
Supplement: Supplementary file 1 — Supplementary Information File [file 41598_2018_32686_MOESM1_ESM.pdf]

Bipolar lophotrichous *Helicobacter suis* combine extended and wrapped flagella bundles to exhibit multiple modes of motility

Maira A. Constantino[1], Mehdi Jabbarzadeh[2], Henry C. Fu[2], Zeli Shen[3], James G. Fox[3], Freddy Haesebrouck[4], Sara Linden[5], Rama Bansil[1, +]

SUPPLEMENTARY INFORMATION

Supplementary Movies:

Movie S1: Flagellar bundle wrapped around the cell. *H. suis* swimming in PGM (15 mg/ml) pH5 imaged with 100X lens, 100 fps.

Movie S2. Inactive extended flagellar bundle and active wrapped flagellar bundle of *H. suis* swimming in BB10 adjusted to pH5 with HCl, imaged with 100X lens, 100 fps.

Movie S3. *H. suis* swimming in a micro-channel formed in PGM (15 mg/ml) pH6, with 100X lens, 343 fps.

Movie S4. *H. suis* transitioning from EE to EW and EW to WW swimming in BB10 adjusted to pH5 with HCl, imaged with 100X lens, 100 fps.

Movie S5. *H. suis* swimming in WW mode in PGM (15 mg/ml) pH6, imaged with 100× lens, 100 fps.

Supplementary Tables:

Table S1: Speed, frequency and shape parameters for all runs of *H. suis*. X_B is the bacterium body axial length, d is the body thickness, N is the number of turns and P is the helical pitch (for a diagram illustrating the body shape parameters see Figure 3A in [14]).

Media	Mode	Run #	α	V (μm/s)	Ω (Hz)	V/ Ω (μm)	X_B^* (μm)	d^* (μm)	N	P^* (μm)
BB10	EW	1	1.99	30 ± 5	36 ± 11	0.8 ± 0.3	6.3	0.9	5	1.3
		2	1.93	18 ± 4	31 ± 12	0.6 ± 0.3	5.5	0.7	4	1.4
		3	1.99	18 ± 3	29 ± 7	0.6 ± 0.2	6.1	0.8	4	1.5
		4	1.77	13 ± 4	33 ± 9	0.4 ± 0.2	6.4	0.8	5	1.3
		5	1.92	32 ± 5	82 ± 17	0.4 ± 0.2	5.1	0.8	4	1.3
		6	2.02	33 ± 12	92 ± 17	0.4 ± 0.2	6.0	0.8	5	1.2
		7	1.86	22 ± 8	38 ± 8	0.7 ± 0.3	7.4	0.8	6	1.2
		8	1.96	21 ± 3	29 ± 7	0.7 ± 0.3	6.8	0.8	5	1.4
		9	2.01	22 ± 2	40 ± 11	0.6 ± 0.2	8.0	0.8	7	1.1
		10	1.98	13 ± 8	33 ± 14	0.7 ± 0.5	10.2	0.8	9	1.1
	EE	11	1.77	6 ± 2	18 ± 2	0.3 ± 0.1	5.5	0.7	6	0.9
		12	1.43	6.9 ± 0.8	42 ± 12	0.2 ± 0.1	6.1	0.8	6	1.0
		13	1.81	7 ± 5	41 ± 12	0.2 ± 0.1	6.1	0.8	6	1.0
		14	1.12	11 ± 6	38 ± 11	0.3 ± 0.2	7.0	0.7	8	0.9
	WW	15	2.01	8 ± 4	30 ± 11	0.3 ± 0.2	6.1	0.8	6	1.0
		16	1.64	7 ± 3	32 ± 7	0.2 ± 0.1	6.4	0.8	6	1.1
		17	1.63	5 ± 2	43 ± 11	0.1 ± 0.1	8.0	0.8	8	1.0
		18	1.38	4 ± 3	4 ± 2	1.0 ± 0.9	8.2	0.8	8	1.0
		19	1.28	3 ± 2	20 ± 6	0.2 ± 0.1	6.7	0.9	6	1.1
PGM	EW	20	1.90	13 ± 4	63 ± 8	0.2 ± 0.1	5.0	0.7	4	1.3
		21	1.98	16 ± 3	19 ± 2	0.8 ± 0.2	5.0	0.6	4	1.3
		22	1.91	19 ± 3	36 ± 13	0.5 ± 0.2	5.0	0.6	4	1.3
		23	2.02	19 ± 2	33 ± 13	0.6 ± 0.3	5.0	0.6	4	1.3
		24	2.01	32 ± 11	78 ± 19	0.4 ± 0.2	5.8	0.7	4	1.5
		25	2.02	27 ± 5	56 ± 10	0.5 ± 0.1	6.4	0.7	5	1.3
		26	1.96	20 ± 3	52 ± 10	0.4 ± 0.1	6.5	0.7	5	1.3
		27	1.99	36 ± 3	45 ± 9	0.8 ± 0.2	6.5	0.7	5	1.3
		28	1.90	34 ± 7	32 ± 13	1.1 ± 0.4	6.5	0.7	6	1.1
		29	1.72	16 ± 13	48 ± 8	0.3 ± 0.7	6.5	0.6	6	1.1
		30	1.89	25 ± 4	43 ± 6	0.6 ± 0.1	6.5	0.6	6	1.1
		31	2.05	29 ± 12	72 ± 21	0.4 ± 0.2	7.0	0.7	6	1.2

		32	1.95	24 ± 3	68 ± 12	0.4 ± 0.1	7.0	0.7	6	1.2
		33	2.01	14 ± 3	47 ± 7	0.3 ± 0.1	8.0	0.7	6	1.3
		34	1.86	11 ± 5	41 ± 10	0.3 ± 0.2	8.0	0.7	6	1.3
		35	1.93	22 ± 4	32 ± 8	0.7 ± 0.2	8.2	0.6	8	1.0
	WW	36	1.80	8 ± 5	43 ± 10	0.2 ± 0.1	7.4	0.5	8	0.9
		37	1.90	11 ± 3	33†	0.33 ± 0.09	7.4	0.5	8	0.9
		38	1.45	8 ± 5	70 ± 21	0.11 ± 0.08	6.5	0.6	7	0.9

* The uncertainty in the measurement is 0.1 μm, given by $\sqrt{2}(pixel\ size)$.

Table S2: Average values of speed, body rotation rate and V/ Ω of *H. suis* swimming in BB10 and PGM for different modes. The average values are given along with standard deviations of measurements.

Media	Mode	V (μm/s)	Ω (Hz)	V/ Ω (μm)
BB10	EW	23 ± 7	45 ± 24	0.6 ± 0.2
	EE	10 ± 2	33 ± 14	0.4 ± 0.2
	WW	7 ± 2	36 ± 9	0.21 ± 0.05
PGM	EW	23 ± 8	48 ± 16	0.5 ± 0.2
	EE	-	-	-
	WW	9 ± 2	45 ± 22	0.2 ± 0.1

Method of Regularized Stokeslets

To investigate the swimming dynamics of *H. suis*, we employ the method of regularized Stokeslets. The surfaces of the cell body and flagella are discretized by regularized Stokeslets which are fundamental solution for the Stokes equation when a localized force is applied to the fluid. The force $\mathbf{f}$ is spread into a "blob" of radius ϵ by $\phi_\epsilon(\mathbf{r}) = 15\epsilon^4\mathbf{f}/\left[8\pi(r^2+\epsilon^2)^{7/2}\right]$, which is an approximation for the Dirac delta function. Using the linear properties of the Stokes flow, the total flow at position $\mathbf{r}$ due to distribution of N regularized Stokeslets is

$$\mathbf{v}(\mathbf{r}) = \sum_{\alpha=1}^{N} \mathbf{S}(\mathbf{r}-\mathbf{r}_\alpha)\mathbf{f}_\alpha \quad (1)$$

where $\mathbf{f}_\alpha$ and $\mathbf{r}_\alpha$ are the force vector and position of the αth regularized Stokeslet, respectively and $S_{ij}(\mathbf{x}) = \frac{1}{8\pi\mu}\left(\frac{\delta_{ij}(\mathrm{x}^2+2\epsilon^2)+\mathrm{x}_i\mathrm{x}_j}{(\mathrm{x}^2+\epsilon^2)^{\frac{3}{2}}}\right)$ is the regularized Stokeslet with blob radius ϵ.

To solve equation (1), for a given geometry, we prescribe a rotation rate of the flagellum with respect to the cell body. Using the linearity of the Stokes equation, we can later scale the results to satisfy conditions on torque or swimming speed. Assuming no-slip

boundary conditions, the velocity at each material point $\mathbf{r}_\alpha$ on the surfaces can be related to the swimming and rotation velocity $(\mathbf{V}, \boldsymbol{\Omega})$ of the cell body in the lab-frame by $\mathbf{v}(\mathbf{r}_\alpha) = \mathbf{V} + \boldsymbol{\Omega} \times \mathbf{r}_\alpha + \dot{\mathbf{L}}_\alpha$, where $\dot{\mathbf{L}}_\alpha = \boldsymbol{\Omega}_\mathrm{f} \times \mathbf{r}_\alpha$ are the prescribed velocities of the Stokeslets on the flagella with respect to the cell body in the body-fixed frame. Thus, the left hand side of equation (1) can be described by six components of the velocity and rotation rate of the cell body. To find these unknowns, we apply force and torque free conditions for the swimmer in the fluid,

$$\sum_{\alpha=1}^{N} \mathbf{f}_\alpha = 0 \,, \qquad \sum_{\alpha=1}^{N} \mathbf{r}_\alpha \times \mathbf{f}_\alpha = 0 \qquad (2)$$

These conditions yields a linear system of equations which can be solved for the six unknowns components of $(\mathbf{V}, \boldsymbol{\Omega})$. Note that as the flagellum rotates, the geometry may change leading to a new solution of $(\mathbf{V}, \boldsymbol{\Omega})$ at each time step as the bacterium swims.

The effects of varying the gap distance on the swimming dynamics in theoretical model

For the wrapped bundle, the gap distance Δ between the surface of the cell body and surface of the filament can change the swimming dynamics. Since we cannot measure the exact gap distance (Δ) from experiments, we perform numerical experiments to study its effects on swimming speeds and cell body rotation rates. These results are shown in figure S1 for the EW mode comparing average swimming speed and body rotations rates for the rod-shaped cell body. Errors are reported relative to results using the reference gap assume that $\Delta = d_f$, which was used for all calculations reported in the main text.

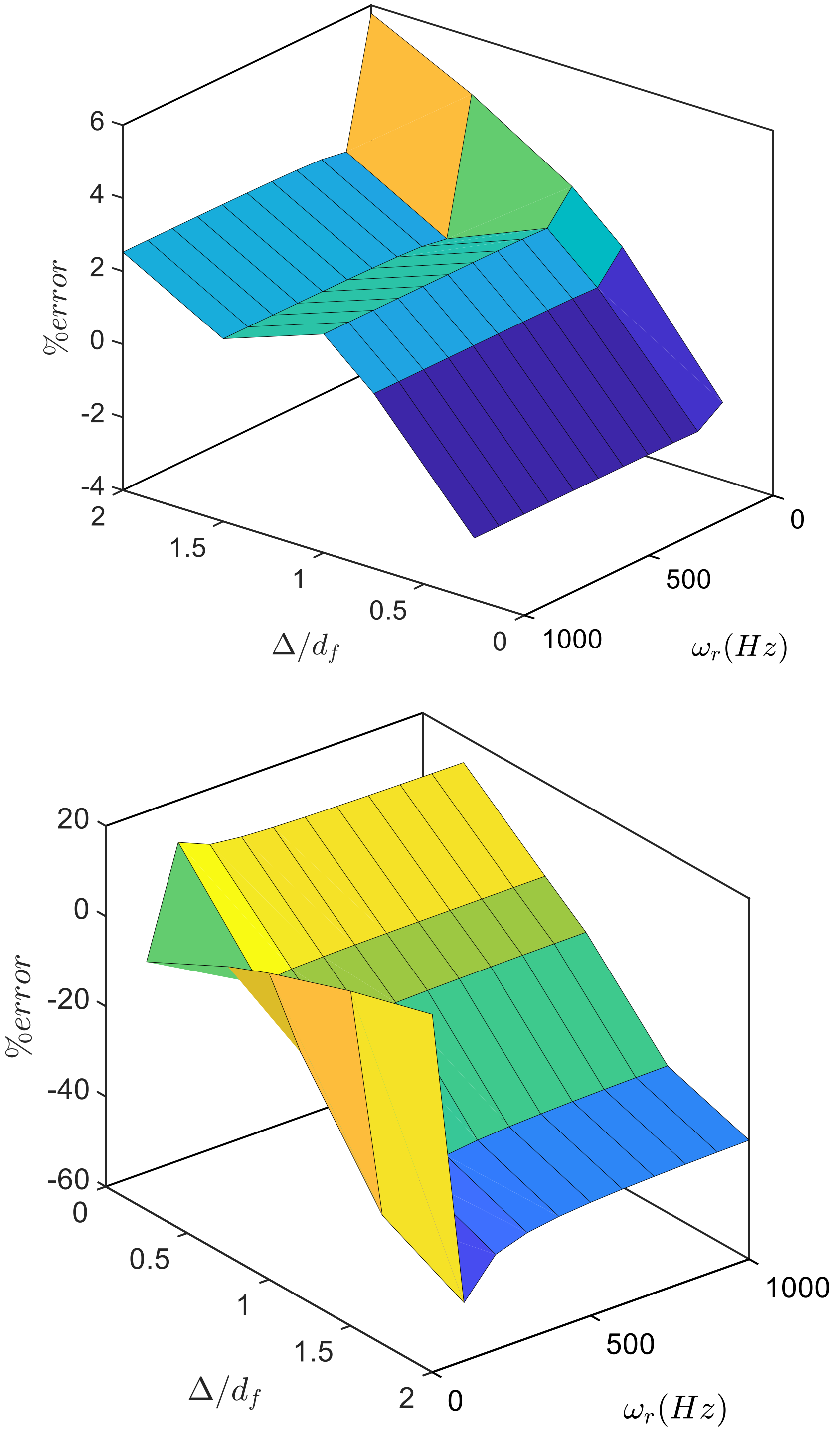

Figure S1: The effects of the gap distance for the average a) swimming velocity and b) for the cell body rotation rate. The gap distance is normalized by the filament diameter and reported errors are relative to results for that $\Delta = d_f$.

Comparing helical cell bodies to the rod shaped cells

In the main text, we used a rod shaped cell body, which allowed us to take advantage of symmetry for the flagella to find velocities and forces in the body frame by combining averaged results calculated for each bundle rotating separately. For the helical cell body, the bacterial geometry changes as each bundle rotates, so to calculate averages we need to average over long times during which both bundles rotate at prescribed rates, and each combination of rotation rates for the two bundles must be calculated separately. Here, we calculate errors in instantaneous translational and rotational speeds for helical and rod shaped cell body in the body frame. For instance, the errors for the EW mode of the helical cell body relative to the rod-shaped body are shown in figure S2.

The instantaneous velocities in the body frame are a function of filament rotation angles (θ_1, θ_2) and relative rotation rates of flagellar filaments $(\omega_1, \omega_2, \omega_r)$. For given rotations angles (θ_1, θ_2), we prescribe the torques to be $T_1 = 2000pN\ nm$ and $T_2 = 2000pN\ nm$ and find instantaneous velocities. We average x-component of translational and rotational velocity of the cell body, over orientation angles (i.e. $\langle V_x \rangle = \int_0^{2\pi} \int_0^{2\pi} V_x(\theta_1, \theta_2)\, d\theta_1 d\theta_2 \,/(4\pi^2)$) to calculate results for helical and rod-shaped cell bodies.

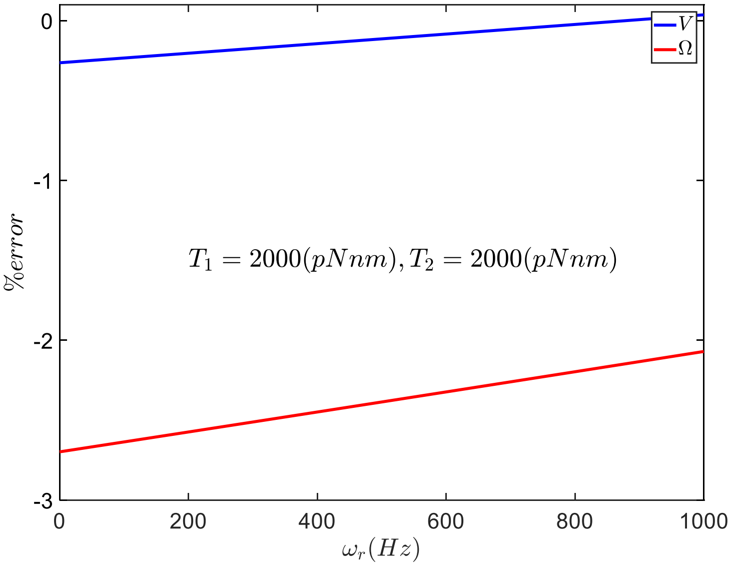

Figure S2: Average errors in comparing the rod-shaped cell body to the helical cell body for the EW case.

The effects of varying the pitch of the wrapped flagellum on the swimming dynamics

In the main text, in the absence of experimental information about the pitch of the wrapped flagellum, we assume that it is same as the extended one. Here, we vary the pitch of the wrapped flagellum for the EW mode to study its effect on the swimming velocities and cell body rotation rates. In Figure S3, we plot swimming velocity and body rotation rates as well as flagella rotation rates for the EW mode as a function of rolling rotation rates ω_r for different pitch ratios P/P_0 where P_0 is the pitch of the extended flagellum given in Table 2. The most effects from varying pitch are in V and W_2 for the higher rolling rotation rate ω_r. For instance for $\omega_r = 100\ Hz$, there are about 10% differences in swimming speed and rotation rate of the wrapped flagellum ω_2 for both pitch ratios of $P/P_0 = 0.5$ and 2 compared to assuming the same pitch as extended flagellum.

Note that the rolling effects are transient, and in the steady-state ($\omega_r = 0$), the effects of varying the pitch of the wrapped flagellum is small for the swimming velocity and cell body rotation rate (about 1%), but we still see about 10% difference in ω_2. To summarize, we expect that our qualitative results are insensitive to the pitch of the wrapped flagellum, with (very conservatively) about 10% uncertainties due to variations in the pitch.

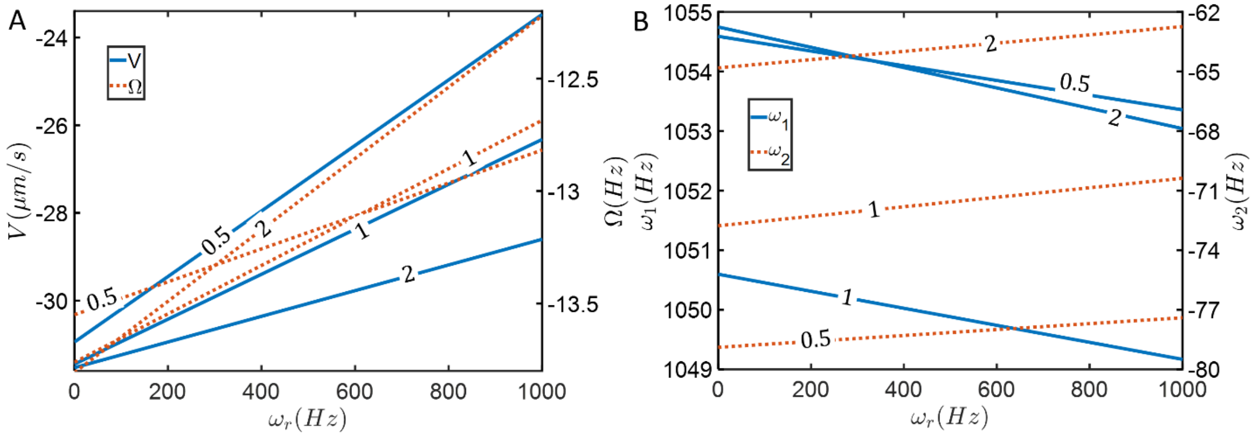

Figure S3: the effects of varying pitch for the wrapped flagellum in EW mode. Labels on the lines show pitch ratio (P/P_0) where P_0 is the pitch of the extended flagellum given in Table 2. We assume constant motor torque of $2000\,pN\,nm$ in x-direction for each flagellum. A) Swimming velocity and cell body rotation rate is plotted as a function of rolling rotation rates ω_r for different pitch ratios. B) Rotational velocities of flagella are shown as a function of rolling rotation rate.
